# Sex-related differences in coordination and variability among foot joints during running

**DOI:** 10.1186/s13047-018-0295-9

**Published:** 2018-09-17

**Authors:** Tomoya Takabayashi, Mutsuaki Edama, Takuma Inai, Masayoshi Kubo

**Affiliations:** Niigata University of Health and Welfare, Institute for Human Movement and Medical Sciences, 1398 Shimami-cho, Kita-Ku, Niigata City, Niigata 950-3198 Japan

**Keywords:** Coordination pattern, Coordination variability, Sex difference, Foot injury

## Abstract

**Background:**

Women, as compared with men, have a higher proportion of injuries in the ankle/foot region. However, the reason for this sex-related difference in foot injuries remains unclear. Recently, joint coordination and variability of coordination have been suggested to be a critical index for defining both the state of injury and the potential risk of injury. The purpose of this study was to investigate sex-related differences in coordination and variability among the foot joints during running.

**Methods:**

Twelve healthy men and 12 healthy women ran on a treadmill. A modified vector coding technique was used to identify coordination and variability among foot joints involving the shank, rearfoot, midfoot, and forefoot segments, and categorized into the following four coordination patterns: in-phase with proximal dominancy, in-phase with distal dominancy, anti-phase with proximal dominancy, and anti-phase with distal dominancy.

**Results:**

There were no differences in all spatiotemporal parameters and in the foot strike angle between men and women. Coordination of variability of the foot joints during running was similar between men and women, but the anti-phase with proximal dominancy in proportion of frontal rearfoot-shank vs. midfoot-rearfoot couple (men; 7.2%, women; 13.9%) and midfoot-rearfoot vs. forefoot-midfoot couple (men; 18.6%, women; 39.8%) in women was significantly increased compared to that in men. Other all coordination of the foot joints during running differed between men and women, and effect sizes of these parameters were all large.

**Conclusion:**

The results may be useful for understanding the underlying mechanism contributing to differences in injury risk in men and women, and may provide novel data on foot joint coordination and variability that could be used as reference data for both biomechanical and clinical running studies.

**Electronic supplementary material:**

The online version of this article (10.1186/s13047-018-0295-9) contains supplementary material, which is available to authorized users.

## Background

The increased awareness of exercise as a necessity for maintaining a healthy lifestyle has made running more popular than ever. While there are many beneficial effects of running, the incidence of running injuries is alarmingly high. One study [1] has suggested that 19.4–79.3% of runners are injured each year. Previous studies [2, 3] have reported a difference in the incidence of ankle/foot injuries between men and women. Frisch et al. [2] investigated the incidence of sports-related injuries in young athletes and found that ankle/foot injuries were the most common in men and women. Furthermore, women had a significantly higher proportion of injuries in the ankle/foot area than men (34.8% and 16.8%, respectively). In particular, overuse injuries of the foot are common among both elite and recreational runners [4]. Among overuse injuries of the foot, plantar fasciitis, which is a common foot injury in runners, was reported in previous study [5] to have an increased prevalence in women. Other researchers [6] have also reported that the incidence of metatarsal stress fractures in women is more than 3.5 times that of men.

Many previous studies [7–10] have investigated traditional kinematic parameters such as range of motion and peak value during dynamic tasks using single joint analysis to identify the reason for the difference between the sexes with respect to the incidence of running injuries. For example, Takabayashi et al. [7] found that women had a greater range of motion and peak values in rearfoot and midfoot motions during running compared to men and reported that this result may provide the reason for the high incidence of foot injuries in women. While potential changes of information on the running injury mechanism have been demonstrated, the motor control mechanism behind these changes is not clear [11]. Additionally, abnormal motion in one joint may influence the kinematics in other joints. However, traditional kinematic parameters cannot simultaneously detect two-joint coordinated relationships.

Other researchers have investigated joint or segment coordination based on vector coding technique, which is a dynamic system approaches and reported that coordination information allows a more sensitive measure of joint mechanism [12]. Altered joint or segment coordination results from a change in either the relative timing or amplitude of motion, and this has been suggested to be a cause of running injuries [13]. For example, compared with healthy runners, injured runners demonstrate altered coordination between the thigh and shank [12]. Moreover, coordination between the shank and rearfoot differs between men and women runners [14]. Taking this into consideration, coordination among the foot joints may be altered between men and women during running, since the incidence of ankle/foot injuries between men and women is different. However, the influence of sex-related differences on coordination among the foot joints remains unclear.

Moreover, variability of coordination has been suggested to be a critical index for defining both the state of injury and the potential risk of injury [15]. In healthy able-bodied individuals, there are many available individual degrees of freedom that can be combined or coordinated to achieve a movement task. Hence, low variability means less flexibility of the body to adapt to changing situations, which may lead to greater repetitive stresses on the lower extremity joints [16]. As the frequently used dynamic system approach for quantifying coordination variability, there is a continuous relative phase in addition to vector coding technique. While the continuous relative phase provides spatio-temporal information from the position and velocity signals, this technique has limitations in quantifying non-sinusoidal couplings and may be not appropriate for most lower extremity couplings during the gait [17]. Thus, quantifying coordination variability is often performed using the vector coding technique in many studies, for example runners with patellofemoral syndrome exhibit less coordination variability than healthy runners by vector coding technique [18]. Moreover, women have decreased variability of coordination at the intra-lower extremity (e.g. between thigh and shank) compared to men [19]. While most of the previous studies have focused on variability of coordination in the intra-lower extremity, it is unknown if or how foot joint coordination variability changes in men and women. By conducting this study, we hope to gain a greater understanding of the underlying mechanism contributing to differences of injury risk in men and women.

The purpose of this study was to investigate sex-related differences in coordination and variability among the foot joints during running. Owing to increased foot injury rates in women [2, 3], we hypothesized that there would be a difference in coordination between men and women, and that its variability would be decreased in women compared to men.

## Methods

### Participants

Twelve healthy men (age = 20.7 (1.2) years; height = 1.71 (0.05) m; weight = 64.8 (9.0) kg) and twelve women (age = 20.4 (1.0) years; height = 1.60 (0.06) m; weight = 52.8 (8.5) kg) participated in this study. Inclusion criteria were as follows: no history of or currently present with lower limb injuries and normal foot based on the arch height index during 90% weight bearing. The reference value for the normal foot is considered to be 1.5 standard deviation (SD) above or below the mean arch ratio measurement of 0.292 (SD 0.027), based on a previously examined sample population of 102 ft [20]. In this study, all participants provided written informed consent before participation. This study was approved by the ethics committee of our institution (No. 17724–160902).

### Experimental protocol

**Fig. 1:**
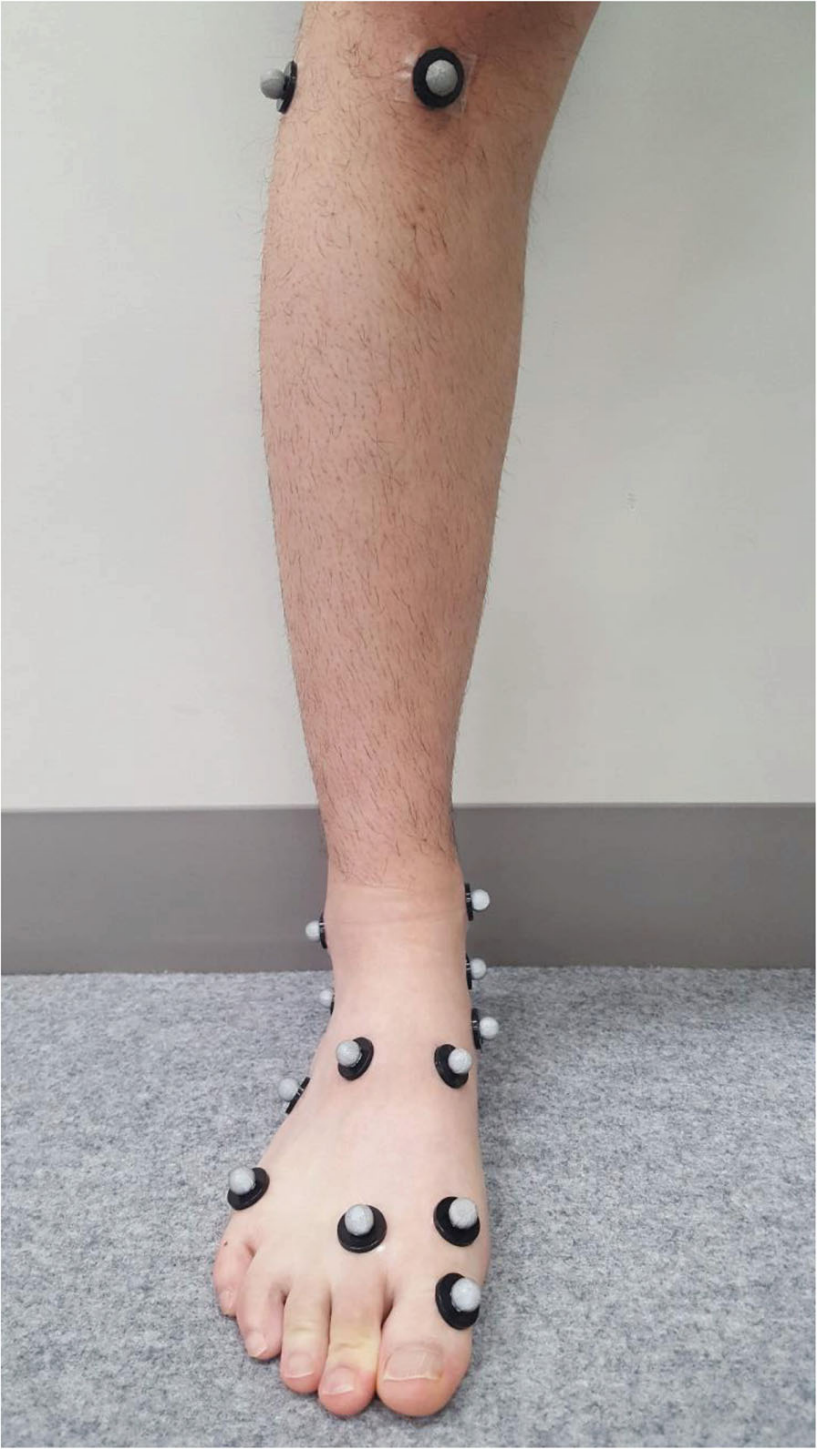
The anterior view of the reflective marker placement on the right shank and foot

Reflective markers of 9 mm in diameter were fixed to the right shank and foot at the tibial tuberosity, fibula head, medial malleolus, lateral malleolus, Achilles tendon attachment, sustentaculum tali, peroneal tubercle, navicular bone, cuboid, first metatarsal base, first metatarsal head, second metatarsal base, second metatarsal head, fifth metatarsal head, and head of the proximal phalanx of the hallux (Fig. 1), according to the Rizzoli foot model [21]. The reproducibility of this model has been confirmed in a previous study [22]. The marker was also attached at the right posterior superior iliac spine to determine the stance phase.

Prior to the acquisition of running data, static standing data were measured for each participant. These data were used to calculate the offset values for all joint rotations in each participant [21]. After the measurement, the participants performed barefoot running (individual’s jogging speed and preferred cadence) on a treadmill (Auto Runner AR-100: Minato Medical Science, Japan). Since women are generally smaller in body size as compared to men, women are likely to increase their step length and cadence parameters. Hence, all spatiotemporal parameters were normalized by the following: speed normalized by the square root of the acceleration of gravity × leg length; cadence normalized by the square root of the leg length/acceleration of gravity; and step length normalized by leg length [23]. Such analysis is often used to investigate sex differences in lower extremity kinematics in previous studies [7, 24].

The participants were allowed to practice the tasks repeatedly for more than one minute on the treadmill. The examiner confirmed with the participants through questioning whether they were accustomed to the motion. After all the subjects verbally reported feeling comfortable running on the treadmill, the task was measured for 30 s. From the strides measured in 30 s, five strides were randomly extracted for each participant. The examiner confirmed the question that all participants had no fatigue prior to measurement of the task or ending the task. The static data and task were measured using a three-dimensional motion analysis system (Vicon, Oxford, United Kingdom) that included 13 infrared cameras at a sampling rate of 250 Hz.

### Data analysis

Raw marker trajectory data during running were filtered using a second-order, zero-lag Butterworth low-pass filter with a 12-Hz cut-off frequency [9]. The following four segments were defined in the kinematics model: the shank comprising the tibia and fibula; the rearfoot (i.e., calcaneus); the midfoot comprising the navicular, cuneiform, and cuboid bones; and the forefoot comprising the first to fifth metatarsal bones. In this study, the three-dimensional joint angles were calculated at the distal segment and expressed relative to the adjacent proximal segment using a right-handed orthogonal Cardan Xyz sequence of rotations (a sequence of plantarflexion/dorsiflexion, eversion/inversion, and abduction/adduction) [25], which was selected to be equivalent of the joint coordinate system [26]. Hence, the joint angles were calculated as plantarflexion/dorsiflexion, eversion/inversion, and abduction/adduction of the rearfoot with respect to the shank (RF-SH), midfoot with respect to the rearfoot (MF-RF), and forefoot with respect to the midfoot (FF-MF).

After calculation of the joint angles during the task, the data were time-normalized to the stance phase (100 data points). The stance phase was determined from the marker trajectory data of the calcaneus, second metatarsal head, and posterior superior iliac spine using Smith’s custom designed algorithm [27]. Briefly, foot strike was defined to occur at the point of maximum in vertical displacement between the calcaneus and posterior superior iliac spine markers. Similarly, toe-off was defined to occur at the point of maximum in vertical displacement between the second metatarsal head and posterior superior iliac spine markers. This method can provide an accurate estimation of foot strike and toe-off. The foot strike angle [28] was also calculated because differences in foot strike angles affect intra-foot kinematics and coupling of the foot joints [29].

### Calculation of the coupling angle and variability

**Fig. 2:**
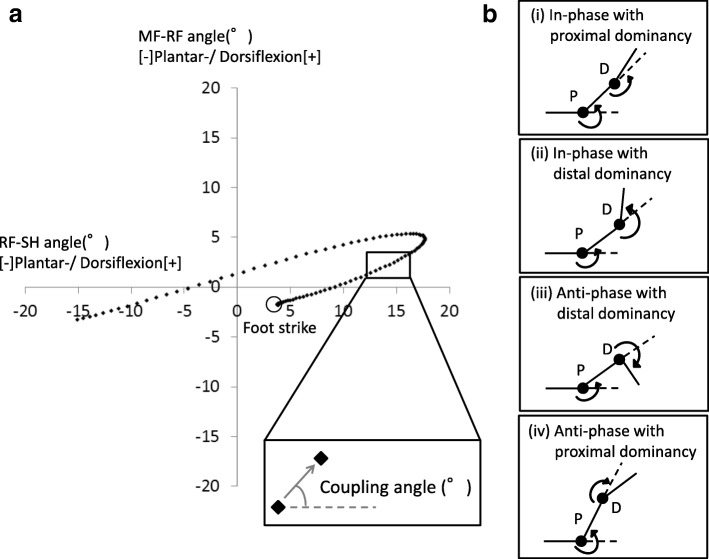
**a** Angle-angle plot of RF-SH and MF-RF. **b** Graphical representation of the four coordination patterns. SH; Shank, RF; Rearfoot, MF; Midfoot, P; Proximal joint, D; Distal joint

Inter-joint coordination was inferred from a coupling angle (*γ*) (Fig. 2a). The coupling angle was calculated using the modified vector coding technique in this study [30].

$$ {\gamma}_{j,i}=\kern0.5em {\tan}^{-1}\left(\frac{y_{j,i+1}-{y}_{j,i}}{x_{j,i+1}-{x}_{j,i}}\right) $$

where 0^°^ ≤ *γ* ≤  360^°^, *x*_i_, and *y*_i_ represent the proximal and distal joint angles, respectively. In addition, *i* represents the percent stance of the *j*th stride. To determine the coupling angle for a single participant (i.e., five strides) and among the participants, the mean coupling angle was calculated from the mean *x*_i_ and the mean *y*_i_ at each percentage of stance. The calculations were performed according to circular statistics [31]. Coordination variability was calculated as the circular SD of the coupling angle for each individual across five strides of data.

The foot joint couplings of interest included the following: (1) sagittal RF-SH vs. sagittal MF-RF, (2) frontal RF-SH vs. frontal MF-RF, (3) sagittal MF-RF vs. sagittal FF-MF, and (4) frontal MF-RF vs. frontal FF-MF. These pairs of joint rotations have been investigated in previous studies [32]. They were chosen to show stronger kinematic coupling during running [33]. The coupling angle represents an instantaneous spatial relationship from which four coordination patterns (Fig. 2b) can be identified: (i) in-phase with proximal dominancy (the same direction and greater angular amplitude of the proximal joint), (ii) in-phase with distal dominancy (the same direction and greater angular amplitude of the distal joint), (iii) anti-phase with proximal dominancy (the opposite direction and greater angular amplitude of the proximal joint), and (iv) anti-phase with distal dominancy (the opposite direction and greater angular amplitude of the distal joint) [34]. In the present study, the positive direction of joint rotation was defined as dorsiflexion and inversion.

**Fig. 3:**
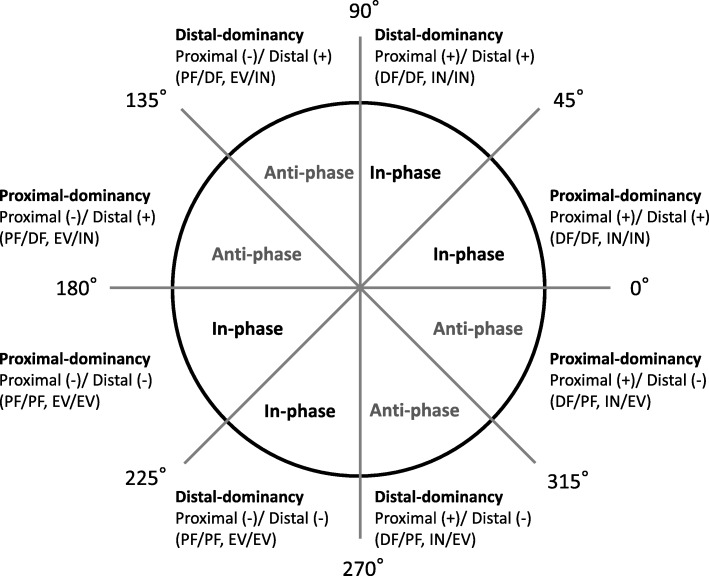
Classification of coordination pattern. The coupling angle was categorized into four coordination patterns. The positive direction (+) represents dorsiflexion (DF), inversion (IN). The negative direction (−) represents plantarflexion (PF), eversion (EV)

There are two functional phases, absorption and propulsion, during the stance phase of running, and the two phases switch at the moment when rearfoot eversion changes to rearfoot inversion [13]. The two phases were divided based on the peak value of rearfoot eversion in each participant. The mean coupling angles were categorized into one of the four coordination patterns at each phase, as shown in Fig. 3. All computations were performed in Scilab version 6.0.0 (Enterprises, Versailles, France).

### Statistical analysis

An independent *t*-test was used to detect sex-related differences among normalized spatiotemporal parameters, foot strike angles, and foot joint coordination patterns in each functional phase. The alpha level for all tests was set at 0.05. Statistics testing was performed using R version 3.3.2 (The R Foundation for Statistical Computing, Austria). Effect sizes (ES) were also calculated by using Cohen’s d statistics. The ES were evaluated on the following criteria: trivial (0–0.19), small (0.20–0.49), medium (0.50–0.79), and large (> 0.80).

## Results

While speed (men: 2.17 (0.12) ms^− 1^, women: 2.04 (0.13) ms^− 1^) and step length (men: 0.78 (0.04) m, women: 0.71 (0.06) m) significantly differed between the men and women (*P* < 0.05), there were no differences in the normalized speed (men: 0.75 (0.04), women: 0.73 (0.04), *P* > 0.05), normalized cadence (men: 49.1 (2.4), women: 49.3 (2.9), *P* > 0.05), normalized step length (men: 0.92 (0.05), women: 0.88 (0.07), *P* > 0.05), and foot strike angle (men: 16.8° (5.1°), women: 15.6° (3.2°), *P* > 0.05) between men and women.

**Fig. 4:**
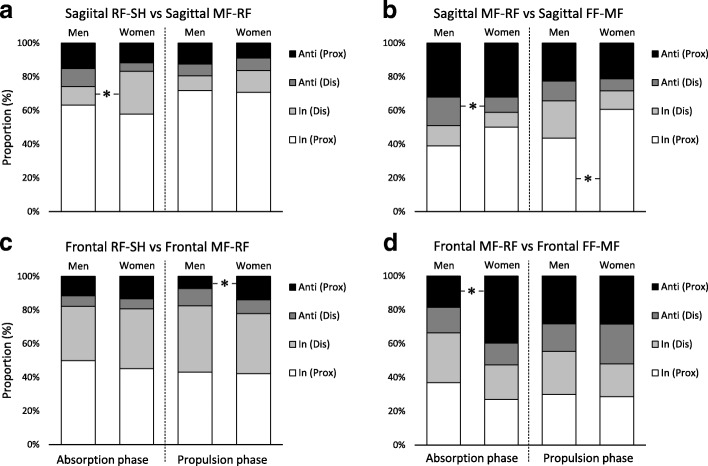
Stacked graph for inter-joint coordination patterns during running. * indicates significant change between sex (*P* < 0.05). In (Prox); In-phase with proximal dominancy, In (Dis); In-phase with distal dominancy, Anti (Dis); Anti-phase with distal dominancy, Anti (Prox); Anti-phase with proximal dominancy, SH; Shank, RF; Rearfoot, MF; Midfoot, FF; Forefoot

In the sagittal plane, RF-SH vs. MF-RF couple, during the absorption phase, women showed a significant increase in the proportion of in-phase with distal dominancy (the same direction and greater angular amplitude of MF-RF) compared with men (men: 11% (6.1%), women; 25.4% (12.3%), Fig. 4a). During the absorption phase, in the sagittal MF-RF vs. FF-MF couple, men showed a significant increase in the proportion of anti-phase with distal dominancy (the opposite direction and greater angular amplitude of MF-RF) compared with men (men; 17.0% (7.4%), women; 9.1% (5.7%), Fig. 4b). In the sagittal MF-RF vs. FF-MF couple during the propulsion phase, women showed a significant increase in the proportion of in-phase with proximal dominancy (the same direction and greater angular amplitude of MF-RF) compared to men (men; 43.6% (21.6%), women; 60.6% (12.0%), Fig. 4b). In the frontal RF-SH vs. frontal MF-RF couple during the propulsion phase, women showed a significant increase in the proportion of anti-phase with proximal dominancy (the opposite direction and greater angular amplitude of RF-SH) compared to men (men; 7.2% (7.8%), women; 13.9% (7.5%), Fig. 4c). In the frontal MF-RF vs. frontal FF-MF couple during the absorption phase, women showed a significant increase in the proportion of anti-phase with proximal dominancy (the opposite direction and greater angular amplitude of MF-RF) compared to men (men; 18.6% (14.3%), women; 39.8% (24.1%), Fig. 4d). The ES of these significant parameters were all large.

**Fig. 5:**
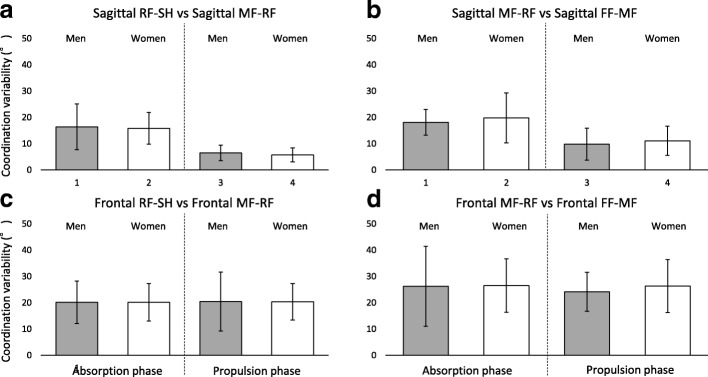
Stacked graph for variabilities of inter-joint coordination patterns during running. SH; Shank, RF; Rearfoot, MF; Midfoot, FF; Forefoot

Variabilities in coordination between the foot joints are shown in Fig. 5. Coordination variabilities for sagittal RF-SH vs. MF-RF, frontal RF-SH vs. MF-RF, sagittal MF-RF vs. FF-MF and frontal MF-RF vs. FF-MF were similar between men and women, and all coordination variabilities were not significantly different (Fig. 5a-d). Moreover, the ES of these parameters were trivial to small. Complete results of coordination and variability are displayed in the Additional file 1: Table S1.

## Discussion

The purpose of this study was to investigate sex-related differences in coordination and variability between the foot joints during running. The results of this study support our first hypothesis that foot joint coordination during running differed between men and women. Contrary to our second hypothesis that coordination variability between foot joints would be lower in women than in men, variabilities in joint coordination were similar between groups.

Some of the coordination patterns were significantly different between men and women (e.g. in-phase with distal dominancy in sagittal RF-SH vs. MF-RF couple). As a rationale for the difference in coordination patterns by sex, DeLeo et al. [13] reported that altered joint or segment coordination results from a change in either the relative timing or amplitude of motion. The coordination changes in this study could also be a result of alternating joint motion amplitudes, alternating joint motion timing, or a combination of both [11].

In many previous studies [11–14, 35], altered joint coordination patterns have been suggested to be a cause or marker of running injury. Chang et al. [30] reported that anti-phase motion in the frontal plane leads to torsion of the soft tissues. It is thought that this motion may overload the soft tissues or joints. Actually, the coordination pattern between trunk and pelvis rotation in the transverse plane in patients with low back pain decreases in the anti-phase and increases in the in-phase compared to controls. This is because patients adjust to avoid or minimize pain [35, 36]. Additionally, sex-related differences in excessive motions of the lower extremities during running have been suggested to be a contributing factor for running injuries [7, 10, 37], for example women showed excessive midfoot movements in the sagittal plane compared to men [7]. The proportion of frontal RF-SH vs. MF-RF and MF-RF vs. FF-MF couples in women was significantly increased in the anti-phase with proximal dominancy. Therefore, the results of this analysis contribute to the factor that women are more likely to have ankle/foot injuries than men. However, this suggestion will require investigation in a longitudinal study.

In this study, all coordination variabilities were not significantly different. Boyer et al. [38] showed that coordination variability in transverse thigh and shank rotation was reduced in women compared to men during running, but those results were inconsistent with the results of this study. However, a previous study [16] reported that the coordination variabilities of the intra-lower extremity were similar between young people and healthy elderly people who have high injury rates. This is due to the fact that those elderly people were healthy, which potentially prevented the injury. Similarly, changes in coordination variability in men and women in this study may have not shown because the target participants were healthy and had no history of disability. Additionally, it was thought to be due to differences in spatiotemporal parameters. Previous studies [14, 39] set the same running speed between men and women. However, because women are generally smaller in body size than men, women are more likely to increase the step length and cadence parameters as compared to men, which may have affected the preferred movement patterns of women. Recent walking and running analysis study [7, 24] has addressed an analysis of sex-related difference by removing the influence of those spatiotemporal parameters. Since the influence of the spatiotemporal parameters was eliminated in this study, coordination variabilities were not significantly different between men and women. Further research into the influence of such factors on variability measures is warranted.

The present study has certain limitations. First, this study was performed with all participants running at a jogging speed because there is a possibility that pain will appear in the foot when barefoot fast running. However, as running kinematics may change when increasing the running speed, the results of this study could have been different under faster running conditions. Second, the present study includes the use of skin-mounted markers to track the underlying skeletal structures. While the Rizzoli foot model used in this study has been validated in an in vitro study [40], this model has not been validated in an in vivo study. Thus, skin markers mounted on externally identifiable bony landmarks in the foot may not follow the underlying individual skeletal segments to be properly evaluated during running. Finally, since this study used a foot model composed of the rearfoot, midfoot, and forefoot, the motion of each foot bone, such as the metatarsals, cannot be investigated. For example, while the forefoot segment in this study comprises the first to fifth metatarsal bones, previous researches on bone pins have reported that there is considerable movement in each metatarsal bone during running and walking [41]. These limitations will need to be addressed in future studies.

## Conclusions

In this study, coordination of variability of the foot joints during running was similar between men and women, but coordination of the foot joints during running differed between men and women. The results of this analysis may be useful for understanding the underlying mechanism contributing to differences in injury risk in men and women. Furthermore, these results provide novel data on foot joint coordination and variability that could be used as reference data for both biomechanical and clinical running studies.

## Additional file

Additional file 1: **Table S1.** Complete results for joint coordination and variability. (XML 7 kb)

## Abbreviations

ES: Effect size
FF-MF: Forefoot with respect to the midfoot
MF-RF: Midfoot with respect to the rearfoot
RF-SH: Rearfoot with respect to the shank
SD: Standard deviation
